# Atomoxetine for the treatment of Attention-Deficit/Hyperactivity Disorder (ADHD) in children with ADHD and dyslexia

**DOI:** 10.1186/1753-2000-3-40

**Published:** 2009-12-15

**Authors:** Calvin R Sumner, Susan Gathercole, Michael Greenbaum, Richard Rubin, David Williams, Millie Hollandbeck, Linda Wietecha

**Affiliations:** 1 Lilly USA, LLC, IN, USA; 2 Department of Psychology, University of York, Heslington, UK; 3 Capstone Clinical Research, Libertyville, IL, USA; 4 Vermont Clinical Study Center, Burlington, VT, USA

## Abstract

**Background:**

The objective of this study was to assess the effects of atomoxetine on treating attention-deficit/hyperactivity disorder (ADHD), on reading performance, and on neurocognitive function in youth with ADHD and dyslexia (ADHD+D).

**Methods:**

Patients with ADHD (n = 20) or ADHD+D (n = 36), aged 10-16 years, received open-label atomoxetine for 16 weeks. Data from the ADHD Rating Scale-IV (ADHDRS-IV), Kaufman Test of Educational Achievement (K-TEA), Working Memory Test Battery for Children (WMTB-C), and Life Participation Scale for ADHD-Child Version (LPS-C) were assessed.

**Results:**

Atomoxetine demonstrated significant improvement for both groups on the ADHDRS-IV, LPS-C, and K-TEA reading comprehension standard and composite scores. K-TEA spelling subtest improvement was significant for the ADHD group, whereas the ADHD+D group showed significant reading decoding improvements. Substantial K-TEA reading and spelling subtest age equivalence gains (in months) were achieved for both groups. The WMTB-C central executive score change was significantly greater for the ADHD group. Conversely, the ADHD+D group showed significant phonological loop score enhancement by visit over the ADHD group. Atomoxetine was well tolerated, and commonly reported adverse events were similar to those previously reported.

**Conclusions:**

Atomoxetine reduced ADHD symptoms and improved reading scores in both groups. Conversely, different patterns and magnitude of improvement in working memory component scores existed between ADHD and ADHD+D patients. Though limited by small sample size, group differences in relation to the comparable changes in improvement in ADHD symptoms could suggest that brain systems related to the therapeutic benefit of atomoxetine in reducing ADHD symptoms may be different in individuals with ADHD+D and ADHD without dyslexia.

**Trial Registration:**

Clinical Trial Registry: ClinicalTrials.gov: NCT00191048

## Background

The 2 most common developmental disabilities of school-aged children are attention-deficit/hyperactivity disorder (ADHD) and learning disabilities, with prevalence rates of 3%-7% and 5%-10%, respectively [1,2]. Of the children diagnosed with learning disabilities, over 80% have a reading disability or dyslexia [3]. Epidemiological and clinical studies suggest that 15%-40% of children with ADHD have concurrent reading disability [4,5]. While these 2 conditions can occur concurrently, the exact nature of the relationship between ADHD and dyslexia is not completely clear. Several studies based on Diagnostic and Statistics Manual of Mental Disorders, Fourth Edition (DSM-IV) criteria report that academic problems and learning disabilities are more common among children with the predominantly inattentive and combined subtypes of ADHD [4]. The impairment in adaptive function conferred independently by ADHD and dyslexia compounds significantly when there are sufficient symptoms to diagnose both conditions. There has also been some speculation based on differential response to ADHD pharmacotherapy that the co-occurrence of ADHD and dyslexia is more related to the inattentive subtype of ADHD, and reduction in hyperactive symptoms alone may not correlate with significant change in reading competency [6].

Both ADHD and dyslexia are also associated with deficits in working memory, the ability to hold information in mind for brief periods of time in the course of ongoing cognitive activities. Low working memory performance has recently been shown to be linked specifically to the severity of inattentive symptoms in ADHD [7] and is also highly characteristic of children with reading difficulties [8,9] Working memory consists of a set of interactive neurocognitive components that include verbal storage, visuo-spatial storage, and the central executive function, which is responsible for regulating task-specific attention [10,11]. Although most studies of working memory function in ADHD and dyslexia have not included assessments of all components of working memory, it has generally been found that both groups show substantial deficits in the central executive function and that an additional impairment of verbal short-term memory may also be present in dyslexia [8].

Atomoxetine hydrochloride (hereafter referred to as atomoxetine) is a nonstimulant, selective norepinephrine transporter inhibitor. Atomoxetine has demonstrated efficacy across age, gender, and ADHD subtypes (inattentive, hyperactive-impulsive, and combined inattentive/hyperactive-impulsive) [12-14]. Specifically, atomoxetine demonstrates efficacy in the predominantly inattentive ADHD subtype [12-14]. For the current study, we evaluated the relative improvement in attention and reading-related benefits, such as more on-task behavior and more consistent information processing. Further, attention; visual and spatial processing; and the use of representational knowledge (working memory) associated to the temporal, parietal, and prefrontal cortex were evaluated [15]. A brief review article of dyslexia reported that neurobiological studies suggest that there may be differences in these areas of the brain in people with dyslexia compared to those who are not reading impaired [16]. Given that ADHD and dyslexia are frequently comorbid, effective treatment with atomoxetine in patients with ADHD and comorbid dyslexia could provide an important treatment advantage without adverse effects on reading performance.

Protocol B4Z-US-LYCE(a) was an open-label, multi-center outpatient, parallel-design, fixed-dose pilot study investigating the efficacy of atomoxetine in reducing symptoms of ADHD in individuals aged 10 to 16 years meeting DSM-IV diagnostic criteria for ADHD and dyslexia. As part of this investigation, a smaller group of individuals meeting DSM-IV criteria for ADHD only were assessed to determine to what extent symptomatic change in the comorbid ADHD and dyslexia group was conferred independently by the effect of atomoxetine on ADHD alone. The primary hypothesis of this study was that atomoxetine would provide therapeutic benefit for the symptoms of ADHD in individuals with ADHD and comorbid dyslexia. Secondarily, the study investigated to what extent any medication-specific change in reading performance may correlate to change in ADHD symptoms and in working memory function, and to what extent the effect of atomoxetine on certain component skills related to reading may correlate with changes in overall reading performance.

## Methods

### Study Design

This open-label, non-randomized, parallel-design pilot study was conducted from October 2003 to March 2006 at 12 centers in the United States. The study protocol was approved and conducted in accordance with the principles of the Declaration of Helsinki, and all parents or legal guardian(s) of the patients provided written informed consent (and patients provided assent where applicable) after the procedure(s) and possible side effects were fully explained. All patients meeting criteria received open-label treatment with atomoxetine at doses ranging from 1.0-1.4 mg/kg once daily given orally as capsules (Strattera^®^, Eli Lilly and Company, Indianapolis, IN, USA) for approximately 16 weeks. The maximum dose prescribed was 1.4 mg/kg or 100 mg, whichever dose was less. After initiating treatment, patients were assessed every 2 weeks for 8 weeks and then once a month for the remaining 8 weeks of the study.

### Patients

Male and female patients, aged 10 to 16 years, meeting the DSM-IV diagnosis of ADHD and/or ADHD with comorbid dyslexia (ADHD+D) were enrolled. In addition to meeting DSM-IV diagnosis criteria for ADHD, patients in the ADHD-only and ADHD+D treatment groups were required to meet Kiddie Schedule for Affective Disorders and Schizophrenia for School-Aged Children-Present and Lifetime, Behavioral Disorders Supplement [17] module criteria for ADHD. Further, during the screening visits, patients were required to have an ADHD symptom severity score at least 1.5 standard deviations above age and gender norms for at least 1 of the diagnostic subtypes (inattentive or hyperactive/impulsive) or the total score for the combined subtype as assessed by the Attention-Deficit/Hyperactivity Disorder Rating Scale-IV-Parent Version: Investigator Administered and Scored (ADHDRS-IV) [18]. Patients diagnosed as ADHD+D were additionally required to have at least a 22-point discrepancy (representing a 1.5 standard deviation discrepancy) between ability (using the highest intelligence quotient score of the Vocabulary [verbal] subtest standard score [SS]; the Matrices [non-verbal/performance] subtest SS; or the IQ composite score on the Kaufman Brief Intelligence Test [K-BIT] [19]) and achievement (reading composite SS on the Kaufman Test of Educational Achievement [K-TEA] [20]). An IQ composite score of ≥80 was required on the K-BIT.

Patients were excluded for any of the following reasons: weight less than 25 kg or greater than 70 kg at study entry; any current or previous diagnosis of bipolar I or II disorder or psychosis; autism, Asperger's syndrome, or pervasive developmental disorder; serious suicidal risk; serious medical illness or clinically significant laboratory abnormalities, hospitalization, or an excluded medication during the course of the study; a history of substance abuse or dependence within the past 3 months (excluding nicotine and caffeine); a positive urine drug screen for any substances of abuse; and treatment with a monoamine oxidase inhibitor within 14 days prior to baseline.

Concomitant medications with primarily central nervous system activity were not allowed. Medications that are strong CYP2D6 inhibitors or substrates were not permitted. Chronic use of cough and cold medications containing pseudoephedrine or the sedating antihistamine diphenhydramine were not allowed. Narcotic use was not permitted unless special circumstances arose (e.g. limited use post-operatively, etc) and approval of the Lilly physician or designee was granted. Patients on methylphenidate or another prescribed stimulant for the treatment of ADHD were required to be stimulant-free 24 hours prior to obtaining baseline measures and to subsequently discontinue medication 1 day prior to the last screening visit before dispensation of study medication.

### Efficacy Measures

The primary objective was to assess the effect of atomoxetine on patients with ADHD+D as measured by the mean change from baseline on the ADHDRS-IV. Secondary measures included comparison between the ADHD and ADHD+D groups on mean change in the ADHDRS-IV total and subscale scores; K-TEA measures (Reading Decoding, Reading Comprehension, Spelling subtests, and Reading Composite scale) [20]; the Working Memory Test Battery for Children (WMTB-C) [15]; and the Life Participation Scale for ADHD-Child Version: Investigator- and Parent-Rated versions (LPS-C) [21]; as well as the correlation between ADHDRS-IV and both the WMTB-C and K-TEA. The efficacy measure, ADHDRS-IV, was assessed at every scheduled visit, whereas the K-TEA and WMTB-C were assessed at approximately every other clinic visit in order to minimize the test/re-test phenomenon. Inter-rater reliability testing of clinicians administering the ADHDRS-IV was performed to ensure consistency among sites. Further, only psychologists experienced with the administration of the educational tests were permitted to administer and score the K-TEA, K-BIT, and WMTB-C.

The K-BIT is a brief, individually administered measure of verbal and non-verbal intelligence. The test is composed of 2 subtests: Vocabulary and Matrices. Vocabulary measures verbal, school-related skills (*crystallized *thinking) by assessing a person's word knowledge and verbal concept formation. Matrices measure non-verbal skills and the ability to solve new problems (*fluid *thinking) by assessing an individual's ability to perceive relationships and complete analogies [19].

The K-TEA is an individually administered measure of the school achievement of children and adolescents in grades 1 through 12. Age equivalents were used for this study, as they provided a more appropriate indicator of improvement for children with disabilities. The K-TEA Comprehensive Form measures reading decoding and comprehension, spelling, and mathematics applications and computation. The Comprehensive Form subtests used for this study were Reading/Decoding, Reading/Comprehension, and Spelling. The sum of the subtest raw scores (Reading Decoding and Reading Comprehension) make up the Reading Composite score, which is transformed to a standard score that indicates age equivalency [20].

The WMTB-C consists of 9 subtests designed to reflect 3 main components of working memory: central executive, phonological loop, and visuo-spatial sketchpad. The frontal regions of both hemispheres of the brain are associated with the central executive functions of coordinating, processing and storage, controlling flow of information through working memory, and attentional control. The 3 central executive (CE) subtests include: Backward Digit Recall, Listening Recall, and Counting Recall. The phonological loop, located in the temporal lobes of left hemisphere, is associated with functions of temporary storage of material in a phonological (sound-based) form, which includes spoken language and both written language and pictures. The 4 subtests designed to measure phonological loop function are: Digit Recall, Word List Matching, Word List Recall, and Non-word List Recall. The visuo-spatial sketchpad, located in the right hemisphere, is associated with functions of storage of materials in terms of visual or spatial features (non-verbal information). Two subtests tap visuo-spatial sketchpad function: Block Recall and Mazes Memory [15].

### Safety

Safety measures recorded at every visit included spontaneously reported treatment-emergent adverse events (TEAEs) and vital signs. Blood for chemistry and hematology laboratories were collected at baseline, after 4, 6, and 10 weeks of treatment, and at the end of the 16-week treatment period. Electrocardiograms were collected at baseline, after 4 weeks of treatment, and at discontinuation of the study.

### Statistical Methods

The primary measure, the determination of significant improvement from baseline on the ADHDRS-IV total score for patients with ADHD+D, was analyzed using a Student's t-test applied to the least squares mean change from baseline score. Change scores were computed for each patient as the difference between the last observation carried forward (LOCF) score and baseline score. The least squares mean change and associated standard error used in the Student's t-test were derived from an analysis of covariance (ANCOVA) model, with terms for diagnostic group, investigator, gender, age, baseline score, and baseline score-by-diagnostic group interaction. All patients with comorbid ADHD+D and at least 1 baseline and 1 post-baseline score were included in the primary analysis.

Between-group changes from baseline to endpoint in efficacy measure variables were analyzed using a fixed-effects ANCOVA model, with terms for diagnostic group, investigator, gender, baseline score, age, and baseline score-by-diagnostic group interaction. Type III sums of squares were used for between-group tests. Changes within diagnostic group were assessed using Student's t-test applied to the least squares mean for the diagnostic group from the ANCOVA model. There were no adjustments made for the number of tests conducted.

Between-group changes in efficacy measure variables over time were analyzed using the relevant contrast from a restricted maximum likelihood repeated measures model, with terms for diagnostic group, investigator, visit, baseline score, diagnostic group-by-visit interaction, and baseline score-by-diagnostic group interaction. This model used the covariance structure that maximizes Schwartz's Bayesian Criterion and the Kenward-Roger method for estimating denominator degrees of freedom. Student's t-test was applied to the least squares mean and standard error to estimate within-group change to endpoint based on the relevant contrast from the diagnostic group-by-visit interaction from this model.

Incidence of categorical response variables were compared across diagnostic groups using Fisher's exact test. Correlations between change and baseline (Visit 1) scores for LPS-C Investigator- and Parent-rated scales were computed to assess the consistency of responses using alternate sources for rating the patient's behavior. Pearson correlation coefficients were calculated to examine the relationships between the changes of selected efficacy measures. Tests were two-tailed.

## Results

### Baseline Characteristics

**Figure 1:**
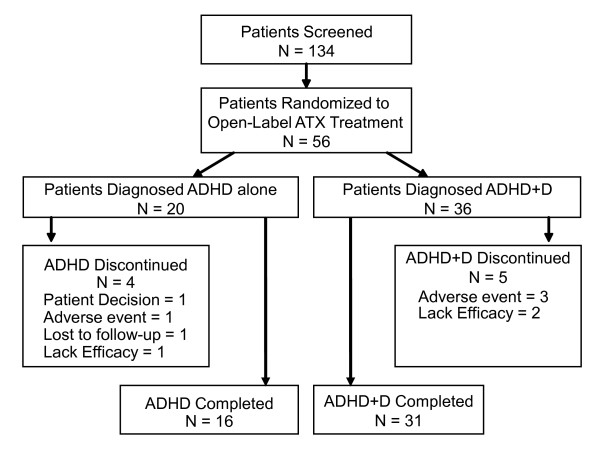
**Disposition of Patients**. Abbreviations: ADHD = attention-deficit/hyperactivity disorder; ADHD+D = ADHD with dyslexia; ATX = atomoxetine.

A total of 134 patients were screened, and 56 patients met criteria for ADHD (n = 20) and ADHD+D (n = 36). A total of 47 patients, 16 in the ADHD group and 31 in the ADHD+D group, completed the study. Figure 1 displays the patient disposition. The patient population for this study was predominantly male (70%), and the median age was 12.6 years and 11.4 years for the ADHD and ADHD+D groups, respectively. Demographic characteristics, including weight, origin, and age, were similar between both groups, with the exception of height. The ADHD subtype was comparable between groups, with most patients diagnosed as predominantly combined (54%) or inattentive (43%) subtype. Whereas the K-BIT IQ composite mean baseline score was comparable between both groups, the ADHD group had a statistically significantly lower K-BIT matrices mean subtest standard score (99.9 ± 13.6) compared with the ADHD+D group (109.5 ± 10.3; p = .004). The K-TEA mean baseline scores were numerically similar between groups (p ≥ .118). The mean final prescribed dose was 1.29 mg/kg/day. Table 1 presents the patient baseline demographics.

**Table 1:** Extension Phase Baseline Patient Characteristics

Characteristic	ADHD (N = 20)	ADHD+D (N = 36)
**Gender**, male, n (%)	15 (75.0)	24 (66.7)
**Age**, years, mean (SD)	12.7 (1.5)	12.2 (2.0)
**Ethnic origin**, n (%)		
Caucasian	14 (70.0)	24 (66.7)
African-American	1 (5.0)	5 (13.9)
Hispanic	4 (20.0)	4 (11.1)
Other	1 (5.0)	3 (8.3)
**ADHD subtype**, n (%)		
Hyperactive/Impulsive	0 (0)	2 (5.6)
Inattentive	9 (45.0)	15 (41.7)
Combined	11 (55.0)	19 (52.8)
**Educational services - placement**, n (%)^a^		
Regular education	7 (35.0)	3 (8.3)
Regular education/Resource room	7 (35.0)	11 (30.6)
Regular education/Special education	4 (20.0)	14 (38.9)
Self-contained special education/Integration	1 (5.0)	5 (13.9)
Self-contained special education/No integration	0 (0)	3 (8.3)
Private school - disabilities	1 (5.0)	0 (0)
**K-BIT**, mean (SD) IQ composite	97.2 (11.9)	102.1 (11.0)

^a ^p-value ≤ .05 for between-group difference.

Abbreviations: ADHD = attention-deficit/hyperactivity disorder group; ADHD+D = ADHD and dyslexia group; K-BIT = Kaufman Brief Intelligence Test; n = sample size; SD = standard deviation.

### Efficacy

**Figure 2:**
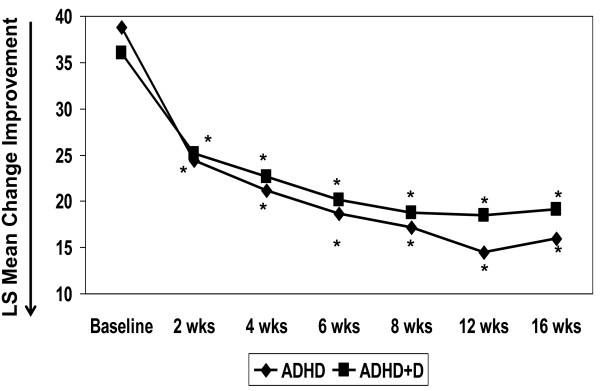
**ADHDRS-IV Total Scores Over 16 Weeks of Treatment**. Abbreviations: ADHD = attention deficit-hyperactivity disorder; ADHD+D = ADHD with dyslexia; ADHDRS-IV = ADHD Rating Scale-IV-Parent Version: Investigator Administered and Scored. * p < .001 for within-group change.

The primary efficacy measure was the ADHDRS-IV total score. Similar statistically significant mean change improvement was demonstrated in both the ADHD and ADHD+D groups on ADHDRS-IV total score (-20.2 ± 2.8 and -17.7 ± 2.5, respectively; p < .001 for both groups) and the subscores for inattention (-11.0 ± 1.6 and -10.4 ± 1.4, respectively; p < .001 for both groups) and hyperactive/impulsivity (-8.5 ± 1.4 and -7.7 ± 1.2, respectively; p < .001 for both groups). There was no differentiation of response between the 2 groups on total score and the inattentive and hyperactive/impulsivity subscores (p = .503, p = .769, p = .660, respectively). Figure 2 presents the ADHDRS-IV total score mean change over time, which further supports improvements in both groups at every time-point throughout the study (p < .001).

The secondary efficacy outcome measure of the LPS-C showed statistically significant adaptive function improvement and agreement between the investigator-rated and parent-rated versions of the scale. Mean change from baseline to endpoint was statistically significant for both groups, though there were no differences between groups.

One of the key secondary objectives in this study was to assess the effects of atomoxetine on K-TEA measures in patients with ADHD or ADHD+D after 16 weeks of treatment. Patients with ADHD and ADHD+D demonstrated statistically significant improvements in mean standard scores and age equivalencies in all K-TEA reading decoding, reading comprehension, reading composite, and spelling measures (p values < .05), with the following exception: The ADHD reading decoding standard score (p = .08) and the ADHD+D spelling standard scores (p = .14) were not statistically significantly improved (Table 2). Baseline measures in the ADHD+D group for reading comprehension and reading composite and spelling measures were numerically lower compared to the ADHD group. The mean change improvements from baseline to endpoint in reading comprehension and reading composite measures were statistically significant for both groups, and there were no statistically significant differences between the groups. The baseline for mean age equivalency (measured in months) was numerically lower for the ADHD+D group for reading comprehension and reading composite compared to the ADHD group. At endpoint, the mean change improvements were statistically significant for both groups. When evaluating improvement by visit, the K-TEA mean reading composite standard score and age equivalency score, reading comprehension standard score, and reading decoding age equivalencies improvements were statistically significant after 4 weeks of treatment and continued to improve after 8 and 16 weeks of treatment for both the ADHD and ADHD+D groups. Improvements in mean reading comprehension standard scores and age equivalencies, and the spelling age equivalencies were statistically significant after 8 weeks of treatment and continued to improve until the end of study for both groups (p ≤ .021). No differences between groups were observed for any of the analyses.

**Table 2:** K-TEA Mean Baseline-to-Endpoint Scores

K-TEA Score, Mean (SE)	Group	Baseline	Endpoint	Change
Reading decoding standard	ADHD	94.3 (9.1)	100.2 (13.5)	3.9 (2.1)
	ADHD+D	80.2 (7.6)	84.8 (10.6)	5.6 (1.8)^a^
Reading decoding age equiv, mo	ADHD	137.7 (30.6)	158.1 (40.9)	17.8 (5.3)^a^
	ADHD+D	104.0 (12.2)	115.5 (22.2)	16.9 (5.7)^a^
Spelling standard	ADHD	90.2 (15.6)	93.0 (16.9)	3.2 (1.1)^a^
	ADHD+D	80.1 (8.6)	82.1 (10.0)	1.5 (1.0)
Spelling age equiv, mo	ADHD	132.6 (40.4)	140.6 (41.6)	9.7 (2.4)^a^
	ADHD+D	106.3 (18.9)	112.1 (25.7)	8.7 (2.2)^a^
Reading comprehension standard	ADHD	98.9 (14.0)	104.0 (15.2)	5.6 (2.0)^a^
	ADHD+D	81.6 (10.8)	89.3 (13.8)	9.8 (1.7)^a^
Reading comprehension age equiv, mo	ADHD	148.4 (41.0)	163.5 (43.8)	17.0 (5.7)^a^
	ADHD+D	106.5 (23.6)	124.8 (35.6)	26.0 (5.2)^a^
Reading composite standard	ADHD	96.6 (11.6)	102.6 (14.9)	4.5 (1.8)^a^
	ADHD+D	80.3 (8.6)	86.4 (11.4)	8.1 (1.6)^a^
Reading composite age equiv, mo	ADHD	144.3 (35.8)	161.9 (40.2)	17.2 (4.4)^a^
	ADHD+D	105.3 (16.6)	120.9 (26.5)	23.5 (4.3)^a^

Last Observation Carried Forward LS Mean Change with no adjustments for number of tests conducted.

^a ^p-value ≤ .05 for within-group changes.

Abbreviations: ADHD = attention-deficit/hyperactivity disorder group; ADHD+D = ADHD and dyslexia group; equiv = equivalent; mo = months; SE = standard error; K-TEA = Kaufman Test of Educational Achievement.

Another key secondary objective evaluated performance on the WMTB-C. For the ADHD group, the mean component and standard scores for central executive function (CE) were statistically significantly improved from baseline to endpoint (p ≤ .032; Table 3). Likewise, the listening recall mean score, a subtest of the CE, was statistically significantly greater from baseline to endpoint (p = .02). Patients with ADHD+D demonstrated statistically significant improvements on the phonological loop (PL) standard score (p = .03) as well as the PL non-word list recall subtest (p = .004). No baseline-to-endpoint improvements were noted for the visuo-spatial sketchpad (VSP) standard or component scores for either group.

**Table 3:** WMTB-C Mean Standard and Component Baseline-to-Endpoint Scores

WMTB-C Score, Mean (SE)	Group	Baseline	Endpoint	Change
**Phonological loop**				
Component score	ADHD	92.4 (12.8)	95.5 (16.2)	1.5 (3.2)
	ADHD+D	90.8 (13.5)	96.7 (14.4)	4.8 (3.0)
Standard score	ADHD	376.0 (39.3)	386.4 (49.8)	5.2 (9.7)
	ADHD+D	365.5 (55.6)	385.5 (54.2)	20.2 (8.9)^a^
**Central executive**				
Component score	ADHD	87.8 (15.7)	97.5 (23.4)	8.4 (3.8)^a^
	ADHD+D	88.3 (13.3)	94.2 (14.5)	4.9 (3.3)
Standard Score	ADHD	268.1 (40.1)	292.8 (51.4)	24.3 (9.8)^a^
	ADHD+D	262.4 (45.7)	270.8 (44.8)	5.9 (9.1)
**Visuo-spatial sketchpad**				
Component score	ADHD	83.9 (16.9)	85.6 (13.1)	0.6 (4.3)
	ADHD+D	87.9 (17.5)	93.2 (20.1)	6.9 (4.1)
Standard score	ADHD	170.3 (33.9)	178.7 (35.1)	6.2 (9.1)
	ADHD+D	162.8 (47.0)	173.8 (50.7)	16.0 (8.5)

Last Observation Carried Forward LS Means Analyses

^a ^p-value ≤ .05 for within-group changes.

Abbreviations: ADHD = attention-deficit/hyperactivity disorder group; ADHD+D = ADHD and dyslexia group; SE = standard error; WMTB-C = Working Memory Test Battery for Children.

**Figure 3:**
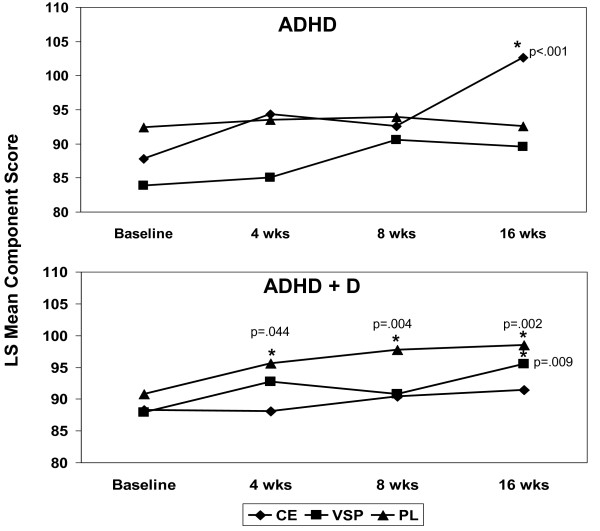
**WMTB-C Component Scores Over 16 Weeks of Treatment**. Abbreviations: WMTB-C = Working Memory Test Battery for Children; ADHD = attention deficit-hyperactivity disorder; ADHD+D = ADHD with dyslexia; PL = phonological loop; CE = central executive; VSP = visuo-spatial sketchpad. * p-value statistically significant.

A by-visit analysis of the least squares (LS) mean changes for the WMTB-C revealed a statistically significantly greater improvement at the last week for patients with ADHD over the ADHD+D group on the CE standard (p = .003) and component scores (p = .012; Figure 3). Subtests of the CE function tests for the ADHD group revealed statistically significant improvements on the listening recall after 8 and 16 weeks of treatment (p ≤ .04) and backward digit recall mean scores at the end of treatment (p = .002). The ADHD group also gained improvements on the PL subtests for non-word list recall after 8 weeks of treatment, which continued to improve until the end of treatment (p < .04). Conversely, patients with ADHD+D demonstrated statistically significant improvement on the PL total standard and component score after only 4 weeks of treatment and continued to improve at every visit throughout the study. PL subtests for the ADHD+D group showed statistically significant gains for the ADHD+D group after 8 weeks of treatment that continued to the end of treatment for word list recall (p < .02) and non-word list recall (p < .01) tests, and statistically significant improvement on the PL digit recall subtest was realized by the end of the study (p < .04). Finally, the baseline-to-endpoint analyses did not reveal significant changes for the VSP component or standard scores for either group. However, the repeated measure analyses (by visit) of the VSP component and standard scores demonstrated statistically significant improvement for patients in the ADHD+D group but only at the end of 16 weeks of treatment (p < .03).

Pearson correlations between the ADHDRS-IV and K-TEA and WMTB-C were calculated. Only the ADHDRS-IV total score, and hyperactivity and inattentive subscores were statistically significantly correlated to the K-TEA Reading Comprehension standard score, though the correlation coefficients were weak (r = .33, r = .31, and r = .28, respectively). There were no statistically significant correlations between the ADHDRS-IV total score or subscores and any other K-TEA measures or to any WMTB-C components.

### Safety

There were no serious adverse events reported. A total of 4 (7.1%) patients of the 56 randomized to the trial discontinued due to adverse events, which included nausea, mood swings, and abdominal pain. Adverse events occurring in at least 5% of all patients in the study were somnolence (n = 19), nausea (n = 17), decreased appetite (n = 12), headache (n = 11), nasopharyngitis (n = 7), upper abdominal pain (n = 11), vomiting (n = 9), cough (n = 4), upper respiratory tract infection (n = 3), constipation (n = 3), irritability (n = 5), psychomotor hyperactivity (n = 3), fatigue (n = 6), and abdominal pain (n = 3). There were no differences between groups in the occurrence of any reported adverse event. Likewise, ECGs, laboratory analytes, vital signs, height, and weight evaluations revealed no clinically significant changes from baseline to endpoint.

## Discussion

These preliminary data demonstrated that atomoxetine was effective in treating ADHD symptoms in patients with ADHD and ADHD+D, and support the primary objective of this study. The presence of dyslexia did not appear to change the response to atomoxetine in reduction of ADHD symptoms. Baseline scores for the academic reading measures for patients in the ADHD+D group were numerically lower, but improvement gains by the end of study were comparable to the ADHD group. Notably, the ADHD+D age equivalent gains of 23.5 months were numerically greater than gains achieved in the ADHD group (17.2 months). Although the K-TEA and WMTB-C were administered 4 times throughout the 16-week study period and therefore could have posed a limitation to the study, the repeated administration of these measures was well within the test/re-test reliabilities described in the test manuals [15,20]. The weakness of correlation between improvements in ADHD symptoms and performance on academic and cognitive measures (i.e. K-TEA and WMTB-C) suggests that the ADHD+D group's academic improvements were not simply a function of improvement of inattentive symptoms. Recent studies of the stimulant methylphenidate in children with ADHD+D and ADHD evaluating response to medication in treatment of ADHD symptoms and reading improvements support the possibility that reading improvements were not likely to be attributed to improvement of inattentive symptoms [22,23].

On measures of neurocognitive function, the baseline values in 3 domains of interest assessed by the WMTB-C were comparable between the 2 groups. The ADHD group showed more marked improvement in the component scores related to central executive function, and the ADHD+D group showed more marked improvement in component scores related to the phonological loop. The phonological, visual-spatial, and central executive tests assess neurocognitive function served by different neural systems. These data could suggest that the brain systems related to the therapeutic benefit of atomoxetine in reducing ADHD symptoms may be different in individuals with ADHD+D and ADHD without dyslexia. The data suggesting that selective areas of working memory can be enhanced by atomoxetine is important, as poor working memory function appears to be a cognitive constraint on academic learning [8].

The findings of this study must be considered in light of design limitations, which include small sample size that was not distributed to groups by randomization and the lack of a placebo comparator group. Although this was an open-label study, the reduction of ADHD symptoms as measured by the ADHDRS was similar to previously conducted placebo-controlled atomoxetine trials [12,13]. Further, this study did not control for special educational services patients may have been receiving upon study entry. However, the actual services used by the study participants were very diverse and unlikely to have played a factor in the results. Given the nature of dyslexia, it would be difficult to limit patients over a 4-month period or disqualify them altogether from receiving special services. Additionally, though it is difficult to make comparisons among non-standardized services, it would be interesting to evaluate whether positive response to medication allowed patients to utilize services more efficiently. Study limitations notwithstanding, the results of this preliminary study provide compelling support that additional benefits may be gained from therapy with atomoxetine in patients with ADHD+D that extend beyond ADHD symptom relief, and that further investigation in larger, placebo-controlled trials is warranted.

## Conclusions

Atomoxetine was equally effective in the reduction of ADHD symptoms for both the ADHD and ADHD+D groups, and both groups saw improvement in reading scores. In contrast, for patients with ADHD and ADHD+D, atomoxetine appeared to provide different patterns and magnitude of improvement in working memory component scores between groups. The data suggests that atomoxetine was well tolerated. Commonly reported adverse events were similar to those reported in previous studies of atomoxetine in children and adolescents [12,24]. Again, this work supports the need for further research in this area.

## Competing interests

CS was a minor stock shareholder and full-time employee of Lilly USA, LLC at the time this manuscript was written. He is currently employed by Biobehavioral Diagnostics Company. DW and LW are minor stock shareholders and full-time employees of Lilly USA, LLC. MH is a former employee of Lilly USA, LLC. SG has no financial conflicts of interest to report. MG has served as a clinical investigator for Eli Lilly and Company and/or one of its subsidiaries, Shire, McNeil/Johnson & Johnson, AstraZeneca, Sanofi-Avenis, Wyeth, Labopharm, Novartis, Abbott, and Pfizer; and has served on speaker's bureaus for Novartis, McNeil, Shire, and Eli Lilly and Company and/or one of its subsidiaries. RR has received research grants from Abbott, Cephalon, Eli Lilly and Company and/or one of its subsidiaries, New River, and Shire; and has served on advisory boards and provided consulting for Addrenex, Cephalon, Eli Lilly and Company and/or one of its subsidiaries, and Shire; and has served on speaker's bureaus for Cephalon, Eli Lilly and Company and/or one of its subsidiaries, and Shire.

## Authors' contributions

CS and LW developed the clinical trial. MG and RR were study investigators. DW was the study statistical expert. All authors contributed to the analysis and interpretation of data. MH drafted the manuscript. All authors critically revised the manuscript for important intellectual content and approved the final version.
